# Analysis of microRNAs in Exosomes of Breast Cancer Patients in Search of Molecular Prognostic Factors in Brain Metastases

**DOI:** 10.3390/ijms23073683

**Published:** 2022-03-27

**Authors:** Carolin J. Curtaz, Leonie Reifschläger, Linus Strähle, Jonas Feldheim, Julia J. Feldheim, Constanze Schmitt, Matthias Kiesel, Saskia-Laureen Herbert, Achim Wöckel, Patrick Meybohm, Malgorzata Burek

**Affiliations:** 1 Department of Gynecology and Obsterics, University Hospital Würzburg, 97080 Würzburg, Germany; curtaz_c@ukw.de (C.J.C.); kiesel_m2@ukw.de (M.K.); herbert_s1@ukw.de (S.-L.H.); woeckel_a@ukw.de (A.W.); 2 Department of Anaesthesiology, Intensive Care, Emergency and Pain Medicine, University Hospital Würzburg, 97080 Würzburg, Germany; reifschlae_l@ukw.de (L.R.); straehle_l@ukw.de (L.S.); constanze.schmitt@icloud.com (C.S.); meybohm_p@ukw.de (P.M.); 3 Center for Translational Neuro- and Behavioral Sciences, University Hospital Essen, 45147 Essen, Germany; jonas.feldheim@uk-essen.de; 4 Department of Neurology, Division of Clinical Neurooncology, University Hospital Essen, 45147 Essen, Germany; 5 Department of Neurosurgery, University Hospital Essen, 45147 Essen, Germany; julia.feldheim@uk-essen.de

**Keywords:** breast cancer, breast cancer metastases, blood-brain barrier, patient serum, exosomes, microRNA, gene expression, prognostic marker

## Abstract

Brain metastases are the most severe tumorous spread during breast cancer disease. They are associated with a limited quality of life and a very poor overall survival. A subtype of extracellular vesicles, exosomes, are sequestered by all kinds of cells, including tumor cells, and play a role in cell-cell communication. Exosomes contain, among others, microRNAs (miRs). Exosomes can be taken up by other cells in the body, and their active molecules can affect the cellular process in target cells. Tumor-secreted exosomes can affect the integrity of the blood-brain barrier (BBB) and have an impact on brain metastases forming. Serum samples from healthy donors, breast cancer patients with primary tumors, or with brain, bone, or visceral metastases were used to isolate exosomes and exosomal miRs. Exosomes expressed exosomal markers CD63 and CD9, and their amount did not vary significantly between groups, as shown by Western blot and ELISA. The selected 48 miRs were detected using real-time PCR. Area under the receiver-operating characteristic curve (AUC) was used to evaluate the diagnostic accuracy. We identified two miRs with the potential to serve as prognostic markers for brain metastases. Hsa-miR-576-3p was significantly upregulated, and hsa-miR-130a-3p was significantly downregulated in exosomes from breast cancer patients with cerebral metastases with AUC: 0.705 and 0.699, respectively. Furthermore, correlation of miR levels with tumor markers revealed that hsa-miR-340-5p levels were significantly correlated with the percentage of Ki67-positive tumor cells, while hsa-miR-342-3p levels were inversely correlated with tumor staging. Analysis of the expression levels of miRs in serum exosomes from breast cancer patients has the potential to identify new, non-invasive, blood-borne prognostic molecular markers to predict the potential for brain metastasis in breast cancer. Additional functional analyzes and careful validation of the identified markers are required before their potential future diagnostic use.

## 1. Introduction

Most cancer-related deaths in women from industrialized nations are caused by breast cancer, which is also the most common malignant tumor in women in the western world. Even though average mortality and overall survival have improved significantly in recent years thanks to innovative new therapies and new and better screening concepts, many patients die prematurely due to a pronounced tumor infestation. Unfortunately, around 10%–15% of all breast cancer patients suffer from brain metastases, resulting in poor overall survival but also a severe impairment in quality of life [1]. The key event for entry into the brain is the migration of cancer cells across the blood-brain barrier (BBB) [2,3]. To date, the exact mechanism of metastatic progression of breast cancer to the brain and migration of cancer cells across the BBB is not well understood in detail [4].

In the search for blood-based factors in breast cancer patients that could impair the integrity of the BBB and thus provoke brain metastases, microRNA (miR) recently came into focus [5]. Only about 20 nucleotides in length, miRs are short, non-coding RNAs that regulate gene expression post-transcriptionally by downregulating mRNA or interfering with its translation [5]. It is known that only 2% of the human genome consists of protein-coding sequences since non-coding sequences predominate [6]. These non-coding sequences are the least studied of the human genome. Their influence on tumor development is not really understood. In recent years, several research groups demonstrated that miRs can be detected in tissues but also in cell-free body fluids such as plasma or serum [7]. In addition, it could be shown that there is a prognostic connection with regard to cancer of different entities [8,9,10].

Extracellular vesicles (EVs) are secreted by eukaryotic cells and can be divided into three categories based on their size: exosomes, activation-or apoptosis-induced microvesicles, and apoptotic bodies. While apoptotic bodies are vesicles with a diameter of only 1–5 µM, microvesicles consist of membrane vesicles and have a diameter of 100–1000 nm. Exosomes, on the other hand, are defined by a diameter of 30–100 nm [11,12]. It was their small size that brought exosomes into the focus of drug delivery but also of biomarker research [13]. They are able to transfer proteins and genetic material [14]. The circulation of exosomes in body fluids allows them to transport a wide variety of active molecules far from their source, where they can absorb and release their contents. Due to their high stability, exosomes are therefore considered to be powerful non-invasive biomarkers [15].

Healthy cells usually release fewer exosomes than tumor cells [16,17]. By analyzing the expression profiles of exosomes isolated from serum/plasma of cancer patients, it can be shown that numerous miRs have different levels compared to healthy individuals [2]. Tumor spread to distant organs of its origin indicated an advanced stage of cancer. Breast cancer is one of the types of cancer that induce metastases in the central nervous system (CNS) with a high incidence, along with lung cancer, melanoma, and colorectal cancer [18]. It could be shown that the isolated exosomes of cancer patients with metastases have different miR expression patterns compared to healthy individuals of patients with primary neoplasia [19]. The development of new and innovative classification criteria for future oncology therapies could help improve tumor therapies, overall patient survival, and quality of life. In connection with the twelfth St. Gallen International Breast Cancer Conference 2011, a classification using biological markers of the primary tumor was introduced [20]. Four subtypes can be defined based on clinical and histological evidence such as expression of estrogen (ER) and progesterone receptors (PR), human epidermal growth factor receptor 2 positivity (HER2), and the proliferation factor Ki-67: luminal A (ER and/or PR+, HER2−, Ki-67 low); HER2− negative luminal B (HER2−, ER and/or PR+, Ki-67 high); HER2− positive luminal B (HER2+, ER and/or PR+, any Ki-67); HER2 overexpressed (any Ki-67, HER2+, ER and PR−, HER2 overexpressed); triple-negative breast cancer (TNBC) (ER and PR−, HER2−). The division into subgroups enables a better differentiation of the individual types in order to make more accurate prognoses and to enable more individual therapy [20].

In the present study, we used TaqMan Advanced miRNA Human Cards to identify the differential expression profiles of miRs in sera from breast cancer patients and to evaluate their prognostic potential. We analyzed the miR levels in the sera of breast cancer patients with primary cancer, bone, visceral or cerebral metastases compared to a healthy control group.

## 2. Results

### 2.1. Patient Characteristics

We collected blood samples from healthy age and sex-matched donors (n = 18), breast cancer patients with primary cancer (n = 15), visceral metastases (n = 18), bone metastases (n = 16) and cerebral metastases (n = 16). The healthy donors had no tumor or a known infection at the time the blood was taken. Clinical data of breast cancer patients were collected for each patient, as shown in Table 1. The median age of patients ranged from 61.1 to 62.9. Most of the patients were postmenopausal. Tumor characteristics and classification of tumors were collected for each patient. Patient serum was stored frozen until use.

### 2.2. Isolation and Characterization of Exosomes Derived from Controls and Breast Cancer Patients with Brain Metastases

**Figure 1:**
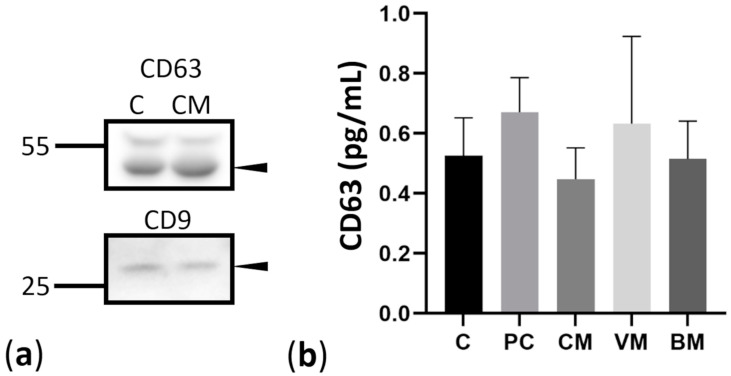
Assessment of exosomal markers. (**a**) Western blot analysis of exosomal markers CD63 and CD9. Arrows indicate the bands corresponding to CD63 (53 kDa) and CD9 (28 kDa), respectively, C—control, CM—cerebral metastases. (**b**) Estimation of exosome levels in patient serum. CD63 protein levels in exosomes from breast cancer patients with primary breast cancer (PC) and breast cancer patients with cerebral (CM), viszeral (VM), and bone (BM) metastases were estimated using a CD63-specific ELISA.

We isolated exosomes from the serum of 83 patients. First, we used two groups of patients, the control group and the group of breast cancer patients with cerebral metastases, to characterize the exosomes in Western blotting to ensure the quality of exosomes. Immunoblotting assay (Figure 1a) revealed the expression of CD63 and CD9, which are widely recognized exosomal-specific markers [21]. Both markers could be detected in isolated exosomes, and there were no differences in levels of both markers between the control and CM groups. We isolated exosomes from 1 mL pooled patient serum as described in methods. Exosomes were characterized for the expression of exosome markers CD9 and CD63 using Western blot (Figure 1a). Protein lysates were prepared out of isolated exosomes, and equal amounts of protein were loaded on gels. CD63 protein was highly expressed in isolated exosomes, while CD9 showed low levels (Figure 1a, arrows). The two analyzed groups of healthy controls and breast cancer patients with cerebral metastasis did not differ in the expression of exosomal markers, although the precipitated pellets differed between the individual samples. Next, we wanted to estimate the amount of exosomes in different patient groups by measuring the CD63 levels at the surface of serum exosomes through a CD63-specific ELISA (Figure 1b). We used sera of healthy controls, breast cancer patients with primary breast cancer, and cerebral, visceral, and bone metastases (Figure 1b). Individual serum samples were measured. Results from ELISA showed a range of 0.5–0.8 pg/mL of CD63-positive exosomes in serum from breast cancer patients with cerebral metastases. The number of exosomes between other groups did not vary significantly by detecting CD63 (Figure 1b).

### 2.3. miRNA Expression in Exosomes Isolated from Patient Serum by Site of Metastasis

**Figure 2:**
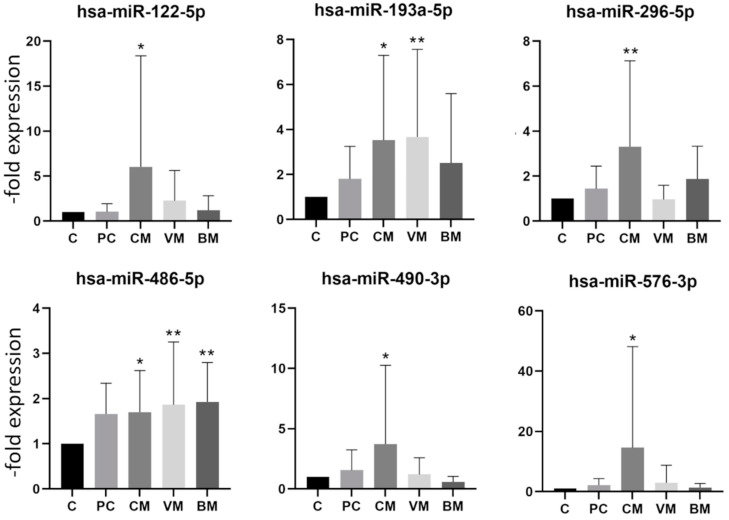
Expression levels of upregulated miRNA in exosomes from healthy controls and breast cancer patients with primary cancer (PC) and cerebral (CM), visceral (VC), or bone (BM) metastases. Data are presented as mean values of fold expression over the control group with standard deviations, * *p* < 0.05, ** *p* < 0.01.

**Figure 3:**
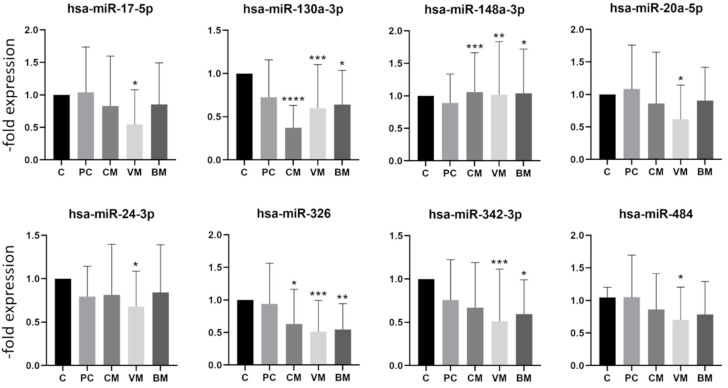
Expression levels of downregulated miRNA in exosomes from healthy controls and breast cancer patients with primary cancer (PC) and cerebral (CM), visceral (VC), or bone (BM) metastases. Data are presented as mean values of fold expression over the control group with standard deviations, * *p*< 0.05, ** *p* < 0.01, *** *p* < 0.001, **** *p* < 0.0001.

In order to first characterize the expression profile of the exosomal miRNA in our samples, we analyzed 384 miRs per sample using the Human TaqMan Advanced miRNA Array Cards A (Thermo Fisher Scientific, Waltham, MA, USA) and the control group and the breast cancer patients with brain metastases. MiRs expressed at levels with Ct values higher than 35 were considered absent from exosomes (results not shown). From the well-expressed miRs, 48 were selected for analysis with a larger patient cohort. Selected miRs and their sequences are presented in Supplementary Table S1. By comparing the exosomal miR expression levels found in serum exosomes of the control group, patients with primary breast cancer, and patients with breast cancer with cerebral, visceral, or bone metastases, several differentially expressed miRs were identified. First, the samples were divided by site of metastasis to the various body regions such as cerebral (n = 16), visceral (n = 18), bone (n = 16), and the group with primary cancer without metastasis (n = 15). The division into four subgroups made it possible to determine whether a specific miR dysregulation is associated with a specific form of metastasis. The comparison of each individual group to the control group revealed a significant difference in the expression of 14 miR in the metastasis groups. Increased expression was identified for six and a decreased expression for eight miRs, as shown in Figure 2 and Figure 3, respectively. None of the six upregulated miRs showed a significant upregulation in all four groups. Hsa-miR-122-5p, hsa-miR-296-5p, hsa-miR-490-3p, and hsa-miR-576-3p were in particular increased in the group with cerebral metastases, while hsa-miR-486-5p showed increased expression in groups with cerebral, visceral, and bone metastases.

Figure 3 depicts the eight miRs with a significantly downregulated expression at least in one of the groups. Again, there was no miR, which was significantly downregulated in all groups. The hsa-miR-130a-3p, hsa-miR-148b-3p, and hsa-miR-326 showed a significant decrease in expression in the group with cerebral, visceral, and bone metastases but were not decreased in the group with primary breast cancer. The hsa-miR-130a-3p showed a particularly strong decrease in the group with cerebral metastases (Figure 3). The fold expression changes of downregulated miRs were not as pronounced as those of upregulated miRs (Figure 2 and Figure 3). Interestingly, none of the analyzed miRs was significantly deregulated in the group with primary breast cancer, which is in accordance with reports showing the involvement of miRs in metastasis development [22].

### 2.4. miRNA Expression in Exosomes Isolated from Patient Serum Classified by Tumor Characteristics

**Figure 4:**
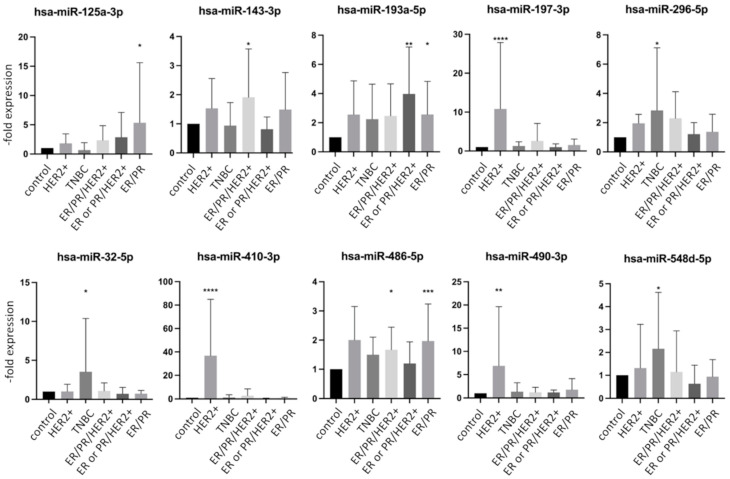
Expression levels of mostly upregulated miRNA in exosomes from healthy controls and breast cancer patients with or without metastases divided by tumor markers. Data are presented as mean values of fold expression over the control group with standard deviations, * *p* < 0.05, ** *p* < 0.01, *** *p* < 0.001, **** *p* < 0.0001.

**Figure 5:**
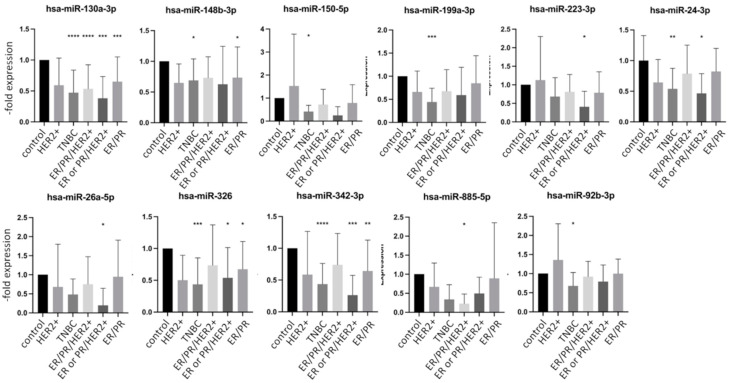
Expression levels of downregulated miRNA in exosomes from healthy controls and breast cancer patients with or without metastases divided by tumor markers. Data are presented as mean values of fold expression over the control group with standard deviations, * *p* < 0.05, ** *p* < 0.01, *** *p* < 0.001, **** *p* < 0.0001.

The expression of different tumor markers is used as a basis for the selection of therapy and correlates with the prognosis. To analyze whether there is a connection between specific miR dysregulation and a receptor status of tumor, we analyzed the same data sets presented in Figure 2 and Figure 3 by sorting the groups by receptor status into five groups: (1) HER2+ (n = 5), (2) TNBC (n = 14), (3) ER/PR/HER2+ (n = 17), (4) ER or PR/HER2+ (n = 7) and (5) ER/PR+ (n = 31). According to previous reports, differential expression patterns of miRs were observed in groups with different tumor markers [20]. When comparing each individual group with the control group, a total of 21 miRs showed a significant dysregulation in the expression pattern. An increased expression profile was found for 10 miRs, while 11 miRs were decreased. Figure 4 and Figure 5 show selected up and downregulated miRs, respectively, in at least one group. Again, none of the examined miRs showed a significant dysregulation in all subgroups simultaneously. The hsa-miR-197-3p, hsa-miR-410-3p, hsa-miR-490-3p showed increased expression in the HER2-positive groups (Figure 4 and Figure 5). The hsa-miR-32-5p was only increased in a TNBC. Hsa-miR-125a-3p was increased in an ER/PR+ group.

### 2.5. Screen of Databases for miR Targets and Signaling Pathways Involved

Next, we looked for targets of the statistically changed miRs and the signaling pathways they are involved in by using TargetScan and KEGG pathway databases as well as HMDD v3.0 (a database for experimentally supported human microRNA-disease associations). The multitude of different targets and signaling pathways could be affected by the far-reaching consequences of miR dysregulation. The validated miR targets are presented in Supplementary Table S2. Due to presented miR dysregulation, metabolic pathways and pathways involved in carcinogenesis are affected. Potential targets involved in mTOR, cAMP, MAPK, HIF1, and p53 signaling can contribute to carcinogenesis and metastasis forming. In addition, exosomal mRNAs show different levels, and their expression can be used as prognostic markers [23]. We analyzed 48 mRNAs that were previously described to be present in exosomes of breast cancer patients [23]. The selected mRNA are shown in Supplemental Table S3. We identified three mRNAs, which were significantly upregulated in serum exosomes of patients with breast cancer with cerebral metastases (Supplemental Figure S1): HSPA5 (heat shock 70 kDa protein 5/binding immunoglobulin protein), FOS (Fos proto-oncogene/AP-1 transcription factor subunit), and LHB (luteinizing hormone subunit beta).

### 2.6. Estimation of Prognostic Potential of Differentially Expressed miRs

**Figure 6:**
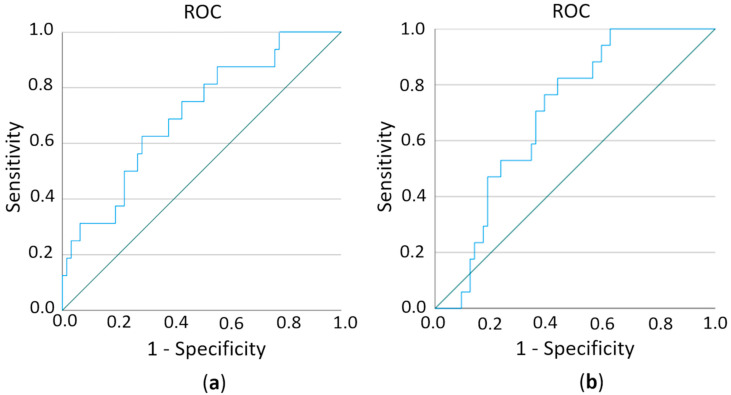
ROC (receiver-operating characteristic) curves of hsa-miR-576-3p (**a**) and hsa-miR-130a-3p (**b**) to test the predictive value of both miRs on brain metastases.

In order to test the sensitivity/specificity of a test criterion, in this case, the predictive value of miR expression on the metastatic pattern of breast cancer patients, we estimated the area under the ROC (receiver-operating characteristic) curve (AUC). ROC curves show the distribution of the expression values within a group. The closer a point is to the upper left corner, the better since that is when sensitivity and specificity are essentially highest. AUC allows a statement to be made as to how well the test criterion (i.e., the respective miR) can predict an assignment to the group (i.e., cerebral metastasis or not). The values between 0.6 and 1 are considered to be predictive. First, we compared the overexpressed miRs with regard to their prediction of the cerebral metastases versus the complete remaining collective (healthy persons, primary breast cancer, and breast cancer with bone and visceral metastases). Here the hsa-miR-576-3p was identified as statistically significant (*p* = 0.012, AUC: 0.705, SD 0.071, 95% CI 0.566–0.844) (Figure 6a). When comparing the downregulated miRs of the cerebral metastasis group versus the complete remaining collective, hsa-miR-130a-3p reached the significance (*p* = 0.012, AUC: 0.699, SD: 0.060, 95% CI 0.582–0.816) (Figure 6b). This regulation (hsa-miR-576-3p upregulated, hsa-miR-130a-3p downregulated) was found in 80% of samples from breast cancer patients with cerebral metastases. This compares to the primary breast cancer group in 19% of the samples, visceral metastases in 21% of the samples, and bone metastases in 20% of the samples.

We analyzed the same data without the control group (healthy control) (cerebral metastases versus primary breast cancer and breast cancer with bone and visceral metastases) since this is closer to the question with potential clinical use of prognostic marker for brain metastases. By analysis of overexpressed miRs, hsa-miR-576-3p was also significant (*p* = 0.048, AUC: 0.666, SD 0.077, 95% CI 0.516–0.816) (data not shown). None of the downregulated miRs was significant in this analysis.

### 2.7. Correlation between Exosomal miRs and Tumor Characteristics

Finally, to verify whether the miRNAs that were differentially expressed in the described series of expression analyses correlate with tumor characteristics, we performed Spearman’s correlation using Spearman’s correlation coefficient. MiRs that correlated statistically with tumor characteristics such as grading and percentage of Ki67-positive cells are presented in Table 2. The levels of exosomal hsa-miR-342-3p correlated inversely with grading.

Further analyses are required to verify the identified miRs as prognostic and diagnostic markers in breast cancer patients for the prevention of brain metastases.

## 3. Discussion

Patient blood can be used to analyze various biomarkers, including miRs [24]. MiRs can regulate gene expression and also contribute to cancer development and progression. Various miRs are dysregulated in several types of cancers. However, the precise contributions of miRs to the molecular mechanisms of breast cancer and, in particular, brain metastases in breast cancer have not yet been fully understood. In this study, we identified two significantly altered miRNA levels when analyzing serum samples from breast cancer patients.

A prerequisite for the analysis of miRNA levels is the isolation of exosomes. Therefore, we used the Total Exosome Isolation Kit to isolate exosomes from serum samples. Other authors have also used the same kit to analyze the miR levels in, e.g., prostate cancer patients [25], samples from patients with focal cortical dysplasia [26], or hepatitis C virus [27]. The authors showed that this method resulted in vesicles with morphology and size compatible with exosomes [25]. The isolated exosomes were smaller than 200 nm and showed an expression of the exosomal markers CD63 and CD9, which we could also detect. The isolation of exosomes from serum or plasma for miR expression profiling has been widely reported. Isolation of exosomes from plasma makes it possible to avoid contamination of circulating exosomes with exosomes that shed platelets during clotting [28], but both methods, isolation from either serum or plasma, provide reliable results [21]. Our analysis is limited to selected miRs only. Other technologies such as miR arrays and small RNA sequencing, RNA-FISH technology, and flow cytometry enable high-throughput detection of all expressed miRs [29,30].

In our results, hsa-miRNA-576-3p is significantly increased in the serum of breast cancer patients with brain metastases. It could be demonstrated that hsa-miR-576-3p targets PD-L1 and cyclin D1 [31]. The PD-1/PD-L1 pathway controls the induction and maintenance of immune tolerance within the tumor microenvironment. The activity of PD-1 and its ligands PD-L1/PD-L2 are responsible for T cell activation, proliferation, and cytotoxic secretion in cancer to degenerating anti-tumor immune responses [32]. D-type cyclins (D1, D2, and D3), along with their associated cyclin-dependent kinases CDK4 and CDK6, are components of the core cell cycle that are responsible for cell proliferation [33]. Hsa-miR-576-3p influencing PD-L1 and cyclin D1 could therefore directly interfere with cancer progression.

Hsa-miR-576-3p has also been identified in association with non-melanoma skin cancer [34]. Its downregulation was observed in plasma samples of non-melanoma skin cancer patients. Other authors identified miR-576-3p as significantly reduced in lung adenocarcinoma, while overexpression of hsa-miR-576–3p in lung adenocarcinoma cells reduced mesenchymal marker expression and inhibited migration and invasion [35]. In addition, the downregulation of hsa-miR-576-3p was observed in bladder cancer tissues and correlated with a poor clinical outcome [36,37]. Those authors concluded, therefore, that hsa-miR-576-3p has a negative regulatory effect on carcinogenesis. In further studies, downregulation of hsa-miR-576-3p was associated with affecting the chemosensitivity of ovarian cancer [31], human teratoma [38], and also breast cancer cells [39].

Our results, in contrast to other published studies, showed a significant increase in hsa-miR-576-3p in serum exosomes from breast cancer patients with brain metastases, while it was at very low levels in all other patient groups, including the control group. Statistical analysis of this miR as a potential prognostic marker revealed a potential of hsa-miR-576-3p as a predictor of cerebral metastases. To the best of our knowledge, this is a first report identifying hsa-miR-576-3p as a potential molecular prognostic factor in brain metastases from breast cancer.

Next to the results of hsa-miR-576-3p, another miR could be identified as a potential molecular prognostic factor. In our statistical analysis, expression of hsa-miR-130a-3p was significantly reduced in cerebral metastases compared to the control group. Hsa-miR-130a-3p seems to have a cancer-promoting function in connection with RAB5B [40]. RAB5B belongs to the Ras family [41]. The RAB protein presumably plays a central role in vesicular transport to the cell membrane but also acts as a tumor-suppressive factor inducing apoptosis and inhibiting angiogenesis [42]. Pan and colleagues could demonstrate in their tumor samples that RAB5A was upregulated and miR-130a was downregulated in breast cancer tissues and cells [43]. Next to that, it could be shown that the endogenous level of RAB5A can be inhibited by the overexpression of hsa-miR-130a [43]. In addition, reduced hsa-miR-130a-3p levels are mentioned in connection with liver fibrosis [44], overexpressed levels in metastatic colon cancer not responding to first-line chemotherapy [45], but also general downregulation in chronic inflammation and macrophagal activity [46]. MDM4, one of the hsa-miR-130a-3p targets, can affect the sensitivity of breast cancer cells to chemotherapy and modulate the p53 signaling pathway [47]. Low expression of hsa-miR-130a-3p correlated with p53 mutation in chronic myeloid leukemia [48]. In breast cancer, the p53 mutation is associated with more aggressive disease and poorer overall survival [49]. A direct influence of hsa-miR-130a-3p on p53 could therefore play a role in the development of brain metastases in breast cancer.

We analyzed the hsa-miR-130a-3p in serum exosomes from breast cancer patients, where it showed significantly reduced levels in the cohort of patients with brain metastases compared to the control group. One reason for this could be that with increasingly reduced hsa-miR-130-3p, the cells become more susceptible to metastatic spread, especially to the CNS. Reduced levels of hsa-miR-130-3p could therefore be a specific prognostic molecular marker for brain metastases in breast cancer.

Changed amounts of miRs can affect the spread and metastasis of visceral tumors. However, the question remains whether and how elevated hsa-miR-576-3p or decreased hsa-miR-130a-3p levels affect the BBB and thus promote its overcoming and brain metastasis formation. Therefore, additional patient samples and molecular studies on in vitro BBB models should be conducted in further investigations. A major problem is the recruitment of patients with brain metastases, as clinically normal patients are not routinely screened for brain metastases [50], while the incidence at autopsy is much higher [51]. There are rarely patients with only brain metastases, as most of them demonstrate visceral metastases first and, in the further course of the disease, the formation of brain metastases. This point is also often discussed critically in data analyses in reviews evaluating published results [22,52]. McGuire states in his review that studies investigating circulating miR profiles in patients with different metastatic diseases should correlate their data to different molecular subtypes of breast cancer [22]. While our study identified hsa-miR-576-3p and hsa-miR-130-3p as prognostic markers for brain metastases in breast cancer, other studies describe hsa-miR-145, hsa-miR-155, hsa-miR-382, and hsa-miR-1910-3p to be present in serum exosomes and promote breast cancer progression [53,54].

Therefore, further analysis is needed to identify the corresponding targets involved in the spread of cancer cells into the CNS.

## 4. Materials and Methods

### 4.1. Patients and Samples

Serum samples were collected from donors after signing an informed consent form in accordance with legislation rules [55]. Ethical guidelines in accordance with the Helsinki Declaration of 1975 and its revision of 1983 were strictly followed.

### 4.2. Exosome Isolation from Patient Serum

Exosomes were isolated as described previously [56]. Briefly, serum samples (0.5–1 mL) were centrifuged at 2000× *g* for 30 min at 4 °C to remove cells and cell debris. A 0.2-fold volume of Total Exosome Isolation (from serum) Kit (Thermo Fisher Scientific) was added for 30 min at 4 °C. The samples were centrifuged at 10,000× *g* for 10 min at room temperature. The resulting pellet, which contained the exosomes, was dissolved in 200 µL of Exosome Resuspension Buffer. Exosomes were frozen at −80 °C or were used immediately for RNA or protein isolation.

### 4.3. Protein Extraction and Western Blot Analysis

Exosomes were extracted as described above. The exosome pellet was shortly washed with 1 mL PBS and centrifuged at 10,000× *g* for 10 min. Exosomes were resuspended in 300 µL Exosome Resuspension Buffer. Protein concentration was estimated using BCA Protein Assay (Thermo Fisher Scientific) following the manufacturer’s instructions. Western blot was performed as previously described [57,58,59]. Briefly, 120 µg of protein extract was mixed with 2× Tris-Glycine Sample Buffer and Reducing Agent (Thermo Fisher Scientific). The samples were loaded on 4%–12% Tris-Glycine Gel (Thermo Fisher Scientific). Separated proteins were transferred to PVDF membrane, blocked with 5% non-fat dry milk for 1 h at room temperature, and then incubated overnight with the primary antibodies anti-CD63 (1:1000; SBI System Biosciences, Palo Alto, CA, USA) and anti-CD9 (1:1000, SBI System Biosciences). After washing the membranes with TBST, incubation with horseradish peroxidase-conjugated anti-rabbit (1:20,000, Cell Signaling, Danvers, MA, USA) was performed for 1 h at room temperature. Next, blots were developed using ECL and FluorChem FC2 Multi-Imager II (Alpha Innotech, San Leandro, CA, USA). The intensity of protein bands was measured with Image J software version 1.52a (NIH, New York, NY, USA).

### 4.4. CD63 Enzyme-Linked Immunosorbent Assay

Enzyme-linked immunosorbent assay (ELISA) was used to measure the CD63 levels on serum exosomes in accordance with the manufacturer’s instructions and as described previously [60,61] (R&D Systems, Minneapolis, MN, USA).

### 4.5. RNA Extraction

RNA was extracted from exosomes using the Total Exosome RNA and Protein Isolation Kit (Thermo Fisher Scientific) according to the manufacturer’s instructions and previous protocols [56]. RNA was eluted in 20–50 µL of DNase/RNase-free water. RNA was quantified using NanoDrop (Thermo Fisher Scientific). RNA was stored at −80 °C until further processing.

### 4.6. cDNA Synthesis and miRNA Expression

The miRNA reverse transcription from 10 ng of RNA extracted from exosomes was performed using the TaqMan Advanced miRNA cDNA Synthesis Kit (Thermo Fisher Scientific) following the manufacturer’s instructions. First, a poly (A) tail was added to one end, and an adapter to the other end of each miRNA and cDNA was synthesized using a universal RT primer, which anneals to the poly (A) tail. Then, each cDNA was preamplified for 14 cycles. The resulting cDNA was frozen for storage at −20 °C. Expression levels of selected miRNAs were investigated using custom-designed TaqMan Advanced miRNA Array Cards (Thermo Fisher Scientific) with 48 miRNAs per sample, including an endogenous control (has-miR-16). Selected miRNAs are reported in Supplementary Table S1. The 48 miRNAs were selected based on the pilot analysis of exosomal microRNA from 18 samples of the control group and breast cancer patients with brain metastases group using Human TaqMan Advanced miRNA Array Cards A (Thermo Fisher Scientific). The most deregulated miRNAs in this analysis were selected, and the analysis groups were expanded to patients with visceral and bone metastases as well as patients with primary breast cancer. Microfluidics cards were run on the QuantStudio 7 flex Fast Real-Time PCR System (Thermo Fisher Scientific). The plates were incubated 10 min at 92 °C for enzyme activation, then amplified in 50 cycles of 1 s at 95 °C denaturation and 20 s at 60 °C annealing/elongation step. QuantStudio^TM^ Real-Time PCR Software (Thermo Fisher Scientific) was used to calculate cycle threshold (Ct) values (cutoff 35 cycles). MiR-320a was used for normalization. Bioinformatic analyses with TargetScan 7.2, the Kyoto Encyclopedia of Genes and Genomes (KEGG) pathway enrichment analysis, and HMDD v3.0 database for experimentally supported human microRNA-disease associations were performed.

### 4.7. Statistical Analysis

Statistical analysis was conducted in GraphPad Prism v9.3 software (GraphPad Software, San Diego, CA, USA). Data are expressed as mean ± standard deviation (SD). Differences among groups were analyzed using the ANOVA with Dunnett’s multiple comparison test. Spearman’s correlation and receiver-operating characteristic (ROC) curves were performed using the IBM SPSS Statistics 23 Software (IBM Corporation, Armonk, NY, USA). *p* values lower than 0.05 were considered statistically significant.

## Figures and Tables

**Table 1:** Patient characteristics. Summary of clinical data.

	C	PC	CM	VM	BM
Patients characteristics					
Total number	18	15	16	18	16
Median age	60.3	61.6	62.9	61.1	61.8
Deceased			12	9	2
Pre-/postmenopausal	4/14	4/11	2/14	3/15	2/14
Tumor characteristics					
ER/PR-positive		8	4	8	10
HER2/neu-positive		5	8	9	5
Triple-negative		2	4	1	1
Grading					
Well differentiated (G1)				1	
Moderately differentiated (G2)		10	9	9	11
Poorly differentiated (G3)		5	7	8	5
% of Ki67-positive cells (median)		22%	43%	34.7%	33.9%
Other			1		

BM = bone metastases, C = control group of healthy donors, CM = cerebral metastases, ER = estrogen receptor, HER2/neu = human epidermal growth factor 2, PC = primary cancer, PR = progesteron receptor, VM = visceral metastases.

**Table 2:** Correlation analysis of miR expression and tumor characteristics.

Correlates With:	Grading	% of Ki67-Positive Cells
% of Ki67-positive cells	r 0.513 ** p < 0.001	
hsa-miR-132-3p	r −0.300 * p 0.043	
hsa-miR-148a-3p	r −0.334 * p 0.022	
hsa-miR-150-5p	r −0.343 * p 0.019	
hsa-miR-197-3p	r −0.368 * p 0.012	
hsa-miR-199a-3p		r −0.339 * p 0.021
hsa-miR-27a-3p	r −0.295 * p 0.044	
hsa-miR-340-5p		r 0.334 * p 0.023
hsa-miR-342-3p	r −0.435 ** p 0.003	
hsa-miR-425-5p	r −0.337 * p 0.021	
hsa-miR-576-3p	r −0.328 * p 0.030	
hsa-miR-885-5p	r −0.312 * p 0.042	
hsa-miR-92b-3p	r −0.297 * p 0.043	

r = correlation coefficient (the positive r means positive correlation, while the negative r means negative correlation; the closer r value to 1 or −1, the greater the correlation); *p* = *p*-value indicating statistical significance, * *p* < 0.05; ** *p* < 0.01.

## Data Availability

Data are contained within the article or Supplementary Material.

## Supplementary Materials

The following are available online at https://www.mdpi.com/article/10.3390/ijms23073683/s1.
