# Supplementary material for: Analysis of microRNAs in Exosomes of Breast Cancer Patients in Search of Molecular Prognostic Factors in Brain Metastases
Source: Int J Mol Sci. 2022 Mar 27;23(7):3683. doi: 10.3390/ijms23073683 (PMC8999078; doi:10.3390/ijms23073683)
Supplement: Supplementary file 1 [file ijms-23-03683-s001.zip › ijms-1644460-supplementary.pdf]

Analysis of microRNAs in exosomes of breast cancer patients in search of molecular prognostic factors in brain metastases

Carolin J. Curtaz [1], Leonie Reifschläger [2], Linus Strähle [2], Jonas Feldheim [3,4], Julia J. Feldheim[5], Constanze Schmitt[2], Matthias Kiesel[1], Saskia-Laureen Herbert[1], Achim Wöckel [1], Patrick Meybohm [2] and Malgorzata Burek [2,*]

Supplementary material

Table S1 miRNAs used in TaqMan Advanced miRNA Array Cards. Next to the miR name is the assay number (Thermo Fisher Scientific).

miRNAs
hsa-miR-122-5p-477855_mir
hsa-miR-125a-3p-477883_mir
hsa-miR-130a-3p-477851_mir
hsa-miR-132-3p-477900_mir
hsa-miR-143-3p-477912_mir
hsa-miR-148a-3p-477814_mir
hsa-miR-148b-3p-477824_mir
hsa-miR-150-5p-477918_mir
hsa-miR-17-5p-478447_mir
hsa-miR-181a-5p-477857_mir
hsa-miR-181b-5p-478583_mir
hsa-miR-185-5p-477939_mir
hsa-miR-193a-5p-477954_mir
hsa-miR-197-3p-477959_mir
hsa-miR-199a-3p-477961_mir
hsa-miR-20a-5p-478586_mir
hsa-miR-210-3p-477970_mir
hsa-miR-214-3p-477974_mir
hsa-miR-21-5p-477975_mir
hsa-miR-222-3p-477982_mir
hsa-miR-223-3p-477983_mir
hsa-miR-23a-3p-478532_mir
hsa-miR-24-3p-477992_mir
hsa-miR-26a-5p-477995_mir
hsa-miR-27a-3p-478384_mir
hsa-miR-296-5p-477836_mir
hsa-miR-29a-3p-478587_mir
hsa-miR-320a-478594_mir
hsa-miR-32-5p-478026_mir
hsa-miR-326-478027_mir
hsa-miR-340-5p-478042_mir

hsa-miR-342-3p-478043_mir
hsa-miR-410-3p-478085_mir
hsa-miR-425-5p-478094_mir
hsa-miR-483-5p-478432_mir
hsa-miR-484-478308_mir
hsa-miR-485-3p-478125_mir
hsa-miR-486-5p-478128_mir
hsa-miR-490-3p-478131_mir
hsa-miR-491-5p-478132_mir
hsa-miR-548d-5p-480870_mir
hsa-miR-576-3p-478164_mir
hsa-miR-598-3p-478172_mir
hsa-miR-625-5p-479469_mir
hsa-miR-652-3p-478189_mir
hsa-miR-885-5p-478207_mir
hsa-miR-92b-3p-477823_mir
hsa-miR-96-5p-478215_mir

Supplementary Table S2: miRNAs and associated targets, signaling pathways and associated diseases (italics: indirect connection, bold: direct connection).

	**Main target genes (TargetScan)**	**Related KEGG pathway**	**HMDD v3.2**
**hsa-miR-122-5p**	**GNG13**, *ALDOA*, PLEKHB2, RFXAP, MASP1	**Pathways in cancer**, *HIF-1 signaling pathway,* Metabolic pathways, Antigen processing and presentation, Coronavirus disease - COVID-19	Stroke, Hepatitis B Virus Infection, Sepsis
**hsa-miR-125a-3p-AL11**	BOK, NIT1, MEAF6, OXLD1, TRAF3IP3	Apoptosis	Rheumatoid Arthritis, Heart Failure, Lung Neoplasms
**hsa-miR-130a-3p**	KDM2A, *MDM4*, KLF7, ENPP5, MYBL1	MicroRNAs in cancer, *p53 signaling pathway*	Thyroid Neoplasms, Muscle Atrophy, Epilepsy
**hsa-miR-143-3p**	C1orf74, ITM2B, SLC25A15, ABHD14A, SLC30A8	Metabolic pathways	Colon Neoplasms, Colorectal Carcinoma, Lymphoma, B-Cell
**hsa-miR-148b-3p**	C18orf25, S1PR1, DCP2, PNRC2, PTPRA	FoxO signaling pathway, Sphingolipid signaling pathway, RNA degradation	Asthma, Anxiety Disorders, Lung Neoplasms

**hsa-miR-150-5p**	MYB, *MDM4*, ENSA, ADIPOR2, HILPDA	MicroRNAs in cancer, *p53 signaling pathway*, Cellular senescence, Renin secretion	Lupus Vulgaris, Sepsis, Human Immunodeficiency Virus Infection
**hsa-miR-17-5p**	GPR6, HAUS8, VSX1, PKD2, NKIRAS1	Taste transduction	Leukemia, Melanoma, Endometriosis
**hsa-miR-193a-5p**	PCDHA7, PCDHA5, CA7, SPRED3, GANAB	Metabolic pathways, ABC transporters	Eosinophilic Esophagitis, Sepsis, Intrahepatic Cholangiocarcinoma
**hsa-miR-197-3p**	CD53, ZIK1, RNF146, USP41, TMEM30C	Herpes simplex virus 1 infection	Diabetes Mellitus Type 2, Myocardial Infarction, Lung Neoplasms
**hsa-miR-199a-3p**	KLHL3, SERPINE2, TMSB4X, PAWR, *NLK*	Regulation of actin cytoskeleton, *Adherens junction, MAPK signaling pathway*	Parkinson Disease, Graft-Versus-Host Disease, Chronic Hepatitis C
**hsa-miR-20a-5p**	GPR137C, CYBRD1, VLDLR, CROT, COX8C	Mineral absorption, Lipid and atherosclerosis, Pathways in cancer, *mTOR signaling pathway*	Leukemia, Carcinoma Hepatocellular
**hsa-miR-223-3p**	FBXW7, RHOB, LELP1, PTS, TBC1D17	Ubiquitin mediated proteolysis, Salmonella infection, RNA degradation	Leukemia, Lymphoblastic, Sepsis, Diabetes Mellitus, Type 2
**hsa-miR-24-3p**	SNN, **FASLG**, REEP2, *STRADB*, NFASC	**Apoptosis**, **Pathways in cancer**, Proteoglycans in cancer, Cell adhesion molecules, *mTOR signaling pathway*	Lung Neoplasms, Pancreatic Neoplasms, Leukemia
**hsa-miR-26a-5p**	*STRADB*, ARPP19, MAB21L1, CREBZF, ZDHHC6	AMPK signaling pathway, *mTOR signaling pathway*	Alzheimer Disease, Diabetic Nephropathy, Chronic Hepatitis B

**hsa-miR-296-5p**	HMGA1, EPN1, RNF44, *TFRC*, TEAD3	Endozytose, *HIF-1-signaling pathway*	Lupus Vulgaris, Atherosclerosis, Hypertension
**hsa-miR-32-5p**	ZFYVE21, PCOLCE2, BTLA, ANP32E, G3BP2		Lung Neoplasms, Colorectal Carcinoma, Viral Infectious Disease
**hsa-miR-326**	KCNIP2, CEP85, CTRC, SYS1, ZNF394		Multiple Sclerosis, Leukemia, Lymphoblastic, Acute; Diabetes Mellitus, Type 1
**hsa-miR-342-3p**	DTNBP1, BTN2A1, RGS4, UBE2D2, MATN1	Protein processing in endoplasmic reticulum	Glioblastoma, Multiple Myeloma, Sepsis
**hsa-miR-410-3p-**	NPPC, DCTN6, CBFB, TRAPPC3, ARFIP1	cGMP-PKG signaling pathway, Vascular smooth muscle contraction, Pathways of neurodegeneration	Stroke, Leukemia, Myeloid, Chronic; Colorectal Carcinoma
**hsa-miR-484-**	HSDL2, DBNDD2, TARBP2, CYB561D1, ARSD		Pancreatic Neoplasms, Metabolic Syndrome, Prostate Neoplasms
**hsa-miR-486-5p-**	GPX8, CSPG5, SRSF3, KIAA0226L, BTAF1	Metabolic pathways, Herpes simplex virus 1 infection	Lupus Vulgaris, Sepsis, Diabetes Mellitus, Type 2
**hsa-miR-490-3p-**	VDAC1, TMOD3, COMMD10, PROSC, HNRNPA1	Alzheimer disease, Influenza A, Spliceosome	Colorectal Carcinoma, Chondrosarcoma, Ovarian Neoplasms
**hsa-miR-548d-5p-**	TBCA, CAMLG, *GHRL*, SRP9, CBWD7	*cAMP signaling pathway*, Protein export	Chronic Hepatitis B, Glioma; Lymphoma, Non-Hodgkin
**hsa-miR-576-3p-**	NPFFR2, ZNF616, MARCH11, GIMAP7, KRTAP21-2	Neuroactive ligand-receptor interaction, Herpes simplex virus 1 infection	Glioma, Melanoma, Bladder Neoplasms
**hsa-miR-885-5p-**	GALNT3, ZNF812, ZADH2, FAM229B, NUDCD2	Metabolic pathways	Pancreatic Neoplasms, Alzheimer Disease, Myasthenia Gravis

**hsa-miR-92b-3p-**	OAZ3, CHCHD10, SERTAD3, MARCH4, NKX2-4	Amyotrophic lateral sclerosis	Heart Failure, Bladder Neoplasms, Gastric Neoplasms

Table S3 mRNAs used in TaqMan Array Cards. Next to the gene symbol, the TaqMan Assay ID is listed (Thermo Fisher Scientific).

mRNA (gene symbol)	TaqMan assay ID
AARS	Hs00609836_m1
ABCC3	Hs02513562_s1
ANP32C	Hs00606537_s1
APOBEC3H	Hs00419665_m1
ATP2A1	Hs01092284_g1
CD9	Hs01124022_m1
CLEC1B	Hs00212925_m1
CLPS	Hs02558461_s1
CSF3R	Hs01116082_g1
DES	Hs00157258_m1
E2F1	Hs00153451_m1
FOS	Hs05634922_s1
GRK2	Hs00176395_m1
HSPA5	Hs00946087_g1
IFNA5	Hs04186137_sH
ITGA2B	Hs01116228_m1
ITGB3	Hs01001469_m1
ITGB5	Hs00174435_m1
KCNH2	Hs00542479_g1
KIF15	Hs01085295_m1
LHB	Hs00751207_s1
MMP14	Hs01037006_gH
MMP9	Hs00957562_m1
MT1E	Hs01938284_g1
MUC4	Hs00366414_m1
MUC5B	Hs06629268_s1
OR3A2	Hs01635424_s1
PAM16	Hs00211426_m1
PCDHGC3	Hs00159905_m1
PILRA	Hs00603661_g1
RILP	Hs00388296_g1
RIN1	Hs00182870_m1
S100A3	Hs00161483_m1
SLC24A3	Hs00915003_m1
SLC35G3	Hs01586570_sH
SLC39A3	Hs00292942_m1

SLC43A1	Hs00992327_m1
SLC7A5	Hs01001187_m1
SPC24	Hs00699347_m1
TGFB1I1	Hs00901623_g1
THBS1	Hs00962908_m1
TNFAIP3	Hs00234713_m1
TNFRSF10C	Hs00182570_m1
TREM1	Hs00218624_m1
TUBB1	Hs00917771_g1
VCX	Hs03645839_gH
VEGFA	Hs00900055_m1

Figure S1

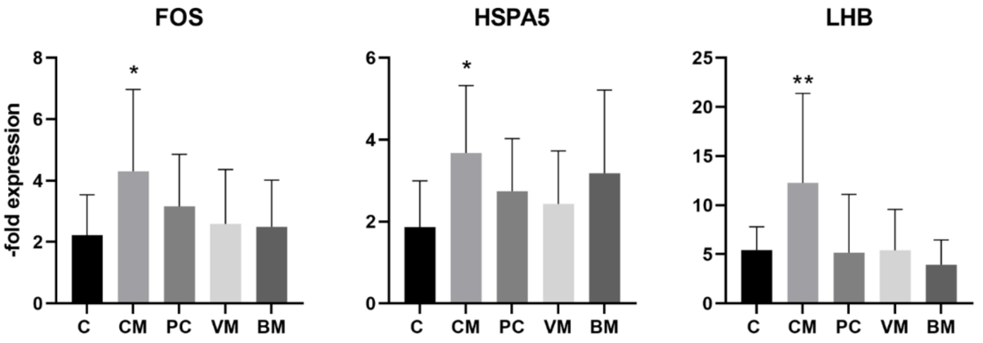

Expression levels of exosomal mRNAs.
